# Assessment of the Impact of Specialized Physical Therapy on the Clinical Condition of Patients After Carpal Tunnel Release

**DOI:** 10.7759/cureus.82529

**Published:** 2025-04-18

**Authors:** Filip Georgiew, Jakub Florek, Adam Bębenek, Grzegorz Sobanski, Pawel Florek, Oles Petrovych

**Affiliations:** 1 Faculty of Health Science, University of Applied Sciences in Tarnów, Tarnów, POL; 2 Department of Orthopaedics and Traumatology, Rydygier Hospital, Brzesko, POL; 3 Department of Neurosurgery, St. Luke Provincial Hospital, Tarnów, POL; 4 Department of Physical Medicine and Rehabilitation, Reha Medica Medical and Rehabilitation Center, Tarnów, POL

**Keywords:** carpal tunnel syndrome, entrapment neuropathy, median neuropathy, postoperative physical therapy, surgical treatment

## Abstract

The aim of this study was to evaluate the impact of specialized physical therapy on the clinical outcomes of patients whose carpal tunnel surgical treatment failed to meet expected results.

The study included 111 patients (130 hands). All patients underwent open carpal tunnel release (CTR) using a standardized surgical technique. Postoperative physiotherapy was uniformly initiated for all patients immediately following the procedure. The effectiveness of treatment was assessed using the Symptom Severity Scale, Functional Status Scale, Numerical Rating Scale, total duration of paresthesia during the provocative test, and sensory excitability threshold evaluation.

The first group consisted of patients who were satisfied with the results of the treatment at the follow-up visit two months after the procedure and who had no complications. This group was not subjected to any additional form of therapy. The second group included patients who expressed dissatisfaction with treatment outcomes during the follow-up visit (two months postoperatively); targeted physical therapy was implemented based on the underlying cause. Statistical analysis showed a statistically significant improvement in all assessed parameters in both groups of patients two months after CTR. The second group exhibited worse outcomes on the symptom severity scale and pain intensity compared to the first group. However, the implementation of specialized physical therapy in this group led to an improvement in clinical condition. By the six-month follow-up, the results achieved in both groups were comparable.

The implementation of additional specialized physiotherapy enhances the clinical outcomes for patients whose carpal tunnel surgical treatment yielded suboptimal results or who encountered postoperative complications. The choice of physiotherapeutic methods should be tailored to the specific symptoms or complications presented by the patient.

## Introduction

Carpal tunnel surgery is the most commonly performed procedure on the upper limb and remains the most effective treatment for carpal tunnel syndrome (CTS) [1]. Its primary advantage is the rapid and permanent resolution of disease symptoms. Carpal tunnel release (CTR) is generally a safe operation, with a major complication rate in those requiring hospital admission or revision surgery of less than 0.1%. Major complications associated with CTR include surgical site infection or dehiscence or neurovascular or tendon injury, requiring admission to hospital or further surgery [2]. However, less severe postoperative issues such as persistent weakness, pillar pain, and scar tenderness are common and can significantly delay recovery and contribute to patient dissatisfaction [3]. According to Sevy and Varacallo, the most frequent complications of CTR include hypertrophic scarring, neuroma formation in the cutaneous branch of the palmar branch of the median nerve, dysesthesia following repeat CTR, wrist stiffness, and incomplete symptom relief [4]. Additionally, Sousa et al. report that complex regional pain syndrome occurs in 2-5% of CTR patients, although most cases resolve within one year [5].

Early postoperative physical therapy is a critical component of comprehensive CTS management, as its absence can diminish or negate the benefits achieved through surgery. Currently, early and "intense" mobilization of the wrist and fingers is emphasized following CTR to facilitate free, longitudinal nerve movement (neuromobilization) and prevent adhesions to adjacent structures [6]. Postoperative physiotherapy typically involves educating patients on proper wound care, scar management, and performing home-based exercises. These exercises often include nerve and tendon gliding, autoneuromobilization of the brachial plexus, and the median nerve tract. However, referrals to specialized hand rehabilitation centers immediately after surgery are less common and are generally reserved for cases where pre-existing symptoms persist or recur during recovery.

The aim of the study was to assess the impact of specialized physical therapy on the clinical condition of patients whose carpal tunnel surgery did not meet expectations.

## Materials and methods

**Figure 1:**
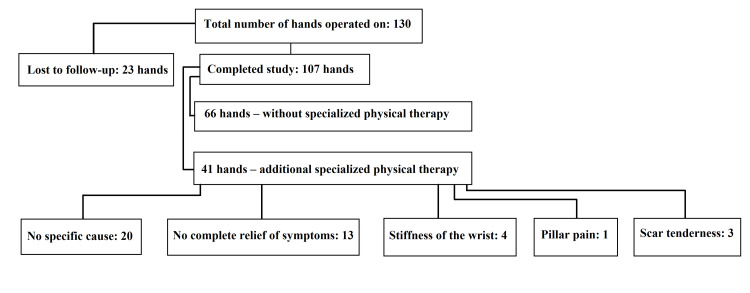
The chart introducing the patient flow throughout the study

The study cohort consisted of 111 patients (130 hands), including 90 women (106 hands) and 21 men (24 hands). A total of 88 patients (107 hands) completed all stages of observation, comprising 70 women (86 hands) and 18 men (21 hands), with an age range of 25 to 77 years (mean age: 54.4 years). Inclusion criteria included CTS confirmed by NCS, symptoms, and clinical examination and informed consent of the patient. Exclusion criteria included patients referred for revision surgery, after fractures of the wrist and distal radius, with rheumatoid arthritis, ulnar nerve syndrome, and diagnosed neurological diseases. The average age of women was 53.5 years, while the average age of men was 58 years (Figure 1). The study was conducted from February 2010 to December 2011 in the Department of Neurosurgery, St. Luke Provincial Hospital, in Tarnów in Poland.

All patients underwent open CTR surgery using a standardized surgical technique. The procedure was performed as a same-day operation under the WALANT (Wide Awake Local Anesthesia No Tourniquet Technique). A longitudinal skin incision was made along the axis of the limb, extending from the distal forearm, approximately 2-3 cm proximal to the wrist crease, into the hand through the thenar groove of the thumb. The median nerve was exposed in the distal forearm, and under direct visualization of the nerve trunk, the dissection was advanced distally. The flexor retinaculum was incrementally incised along its entirety, ensuring it was fully released above the median nerve. The procedure concluded with skin closure, and meticulous hemostasis was achieved using bipolar cautery, avoiding coagulation near the median nerve trunk.

All patients commenced a standardized physiotherapy program starting on the second postoperative day. Following instructions provided by a physiotherapist, the patients performed the exercises independently at home. The program comprised gliding exercises for the flexor tendons and median nerve, self-mobilization of the brachial plexus, therapy focused on improving grip strength and manipulation skills, activities of daily living training, sensory re-education, and wound and postoperative scar management therapy.

All patients were divided into two groups. The first group consisted of patients who expressed satisfaction with the results of the treatment at the follow-up visit (in this group the treatment was completed at this stage). The second group included patients who expressed dissatisfaction with treatment outcomes during the follow-up visit (two months postoperatively); targeted physical therapy was implemented based on the underlying cause. A detailed list of the physiotherapy treatments is presented in Table 1.

**Table 1:** Targeted physical therapy based on the underlying cause

Underlying cause	Physiotherapy treatments
No specific cause	Laser biostimulation, pulsed high-frequency magnetic field therapy, and TENS (Transcutaneous Electrical Nerve Stimulation)
Incomplete symptom relief	Neuromobilization, manual therapy, warm compresses, and laser biostimulation
Wrist stiffness	Whirlpool massage and manual therapy
Scar tenderness	Whirlpool massage, laser biostimulation, iontophoresis with a 2% potassium iodide solution or phonophoresis, and manual scar hardening therapy
Pillar pain	Temporary restriction of hand use and/or immobilization with a brace, warm compresses, and classic massage

Individual parameters were assessed at three time points: before the procedure and two and six months postoperatively. The Levin Questionnaire (also called Boston Carpal Tunnel Syndrome Questionnaire) consisting of two parts was used to assess the intensity of CTS symptoms: Symptom Severity Scale (SSS - assessment of symptom intensity) and Functional Status Scale (FSS - evaluation of hand functionality) [7]. Pain intensity was assessed using the Numerical Rating Scale (NRS). The next parameter used to assess the treatment results was total paresthesia time, measured during the Phalen test, manual compression test (Durkan test), and transverse carpal ligament (TCL) stretching test. The cumulative onset time of CTS symptoms during these tests was recorded. To assess the sensory excitability threshold, we used our own method, which uses pulsed electric current applied to the pulp of fingers I, II, and III, with a pulse duration of 100 ms. The excitability threshold was measured in milliamps. Sensory assessments were conducted twice: before the procedure and six months postoperatively (no evaluation was performed at two months).

## Results

The results presented in Table 2 indicate that, in the group not receiving specialized physical therapy, a statistically significant improvement was observed in all assessed parameters: SSS, FSS, NRS, time of paresthesia, and surface sensation threshold. This improvement was consistent across both observation periods, namely, the first two months and the subsequent four months following surgery. However, this trend did not apply to the surface sensation threshold, which was evaluated only before surgery and at six months postoperatively.

**Table 2:** Changes in the values of the assessed parameters in individual observation periods in the group not using specialized physical therapy SSS: Symptom Severity Scale; FSS: Functional Status Scale; NRS: Numerical Rating Scale; PT: provocative tests; TS: threshold of sensory; CTR: carpal tunnel release 0: before CTR, 2: two months after CTR, 6: six months after CTR

Parameter	Average value	Standard deviation	p-value
Difference SSS 0 – SSS 2	1.35	1.16	p<0.001
Difference SSS 2 – SSS 6	0.49	0.83	p<0.001
Difference FSS 0 – FSS 2	0.89	0.75	p<0.001
Difference FSS 2 – FSS 6	0.45	0.63	p<0.001
Difference NRS 0 – NRS 2	5.0	2.12	p<0.001
Difference NRS 2 – NRS 6	2.19	3.14	p<0.005
Difference PT 0 – PT 2	39.61	55.27	p<0.001
Difference PT 2 – PT 6	22.57	44.07	p<0.001
Difference TS 0 – TS 6	0.27	0.57	p<0.005

In the second group (patients who used additional specialized physical therapy), a statistically significant improvement was also observed in all assessed parameters and observation periods. Detailed results obtained in this group are presented in Table 3.

**Table 3:** Changes in the values of the assessed parameters in individual observation periods in the group using additional specialized physical therapy SSS: Symptom Severity Scale; FSS: Functional Status Scale; NRS: Numerical Rating Scale; PT: provocative tests; TS: threshold of sensory; CTR: carpal tunnel release 0: before CTR; 2: two months after CTR; 6: six months after CTR

Parameter	Average value	Standard deviation	p-value
Difference SSS 0 – SSS 2	0.43	0.82	p<0.001
Difference SSS 2 – SSS 6	1.1	1.01	p<0.001
Difference FSS 0 – FSS 2	0.63	0.85	p<0.001
Difference FSS 2 – FSS 6	0.55	0.71	p<0.001
Difference NRS 0 – NRS 2	2.46	2.93	p<0.001
Difference NRS 2 – NRS 6	3.41	2.86	p<0.001
Difference PT 0 – PT 2	46.58	48.04	p<0.001
Difference PT 2 – PT 6	12.19	33.65	p<0.005
Difference TS 0 – TS 6	0.34	0.34	p<0.001

The comparison of the results for the assessed parameters between the two groups reveals statistically significant differences. Notably, these differences were observed on the SSS and the NRS for pain. At baseline, the initial symptom intensity on the SSS scale was significantly higher in the group that did not use specialized physical therapy (mean = 3.33) compared to the group that did (mean = 2.87), with a p-value of 0.003. After two months of observation, the difference between the groups remained statistically significant, with values of 1.98 and 2.44, respectively (p = 0.02). By the end of the six-month observation period, following the conclusion of physical therapy in group 2, no statistically significant differences were observed between the two groups (p > 0.05). This suggests that additional physical therapy effectively reduced the intensity of symptoms in the group of patients who had not experienced satisfactory improvement after the initial two months (Table 4).

**Table 4:** Comparison of the assessed parameters in individual observation periods in the group using and not using specialized physical therapy SSS: Symptom Severity Scale; FSS: Functional Status Scale; NRS: Numerical Rating Scale; PT: provocative tests; TS: threshold of sensory; CTR: carpal tunnel release; NS: no significance 0: before CTR; 2: two months after CTR; 6: six months after CTR

Parameter	Specialist physiotherapy				p-value
	No (Group 1)		Yes (Group 2)		
	Average value	Standard deviation	Average value	Standard deviation	
SSS 0	3.33	0.77	2.87	0.76	p<0.005
SSS 2	1.98	1.03	2.44	1.03	p<0.05
SSS 6	1.49	0.65	1.34	0.47	NS
FSS 0	2.91	0.81	2.66	0.79	NS
FSS 2	2.02	0.72	2.03	0.96	NS
FSS 6	1.57	0.59	1.47	0.49	NS
NRS 0	7.61	1.43	7.24	1.44	NS
NRS 2	2.12	1.87	4.78	2.71	p<0.001
NRS 6	1.26	2.08	1.36	2.09	NS
PT 0	89.39	44.72	90.97	46.73	NS
PT 2	129.09	47.19	137.56	41.76	NS
PT 6	151.67	44.05	149.76	42.75	NS
TS 0	2.49	0.69	2.47	0.63	NS
TS 6	2.22	0.53	2.13	0.48	NS

Regarding pain intensity, a statistically significant difference was noted on the NRS scale two months after surgery. The first group reported a mean value of 2.12, while the second group reported 4.78. The greater pain intensity observed in the second group prompted patients to seek additional physical therapy. Similar to the symptom severity findings, no statistically significant differences were found between the groups after six months of observation (p > 0.05), indicating that additional physical therapy successfully alleviated pain intensity in patients who did not report adequate improvement in the second month postoperatively (Table 4).

## Discussion

A review of the literature on CTR clearly shows that in the majority of cases, surgical treatment yields excellent results and provides long-lasting relief from symptoms. However, as with any surgical intervention, there is a small subset of patients for whom the treatment outcomes are unsatisfactory, typically due to factors such as incorrect diagnosis or improper patient selection for the procedure. In these cases, patients may experience postoperative complications such as swelling, stiffness, and hyperesthesia of the surgical scar. These patients, in particular, should be considered for additional specialized physiotherapy. The poorer outcomes observed in some patients during the follow-up examination (two months postoperatively) may stem from various factors, including the persistence of CTS symptoms, heightened pain sensitivity, a desire for prolonged sick leave, or the need for further health services. Additionally, minor postoperative complications such as healing difficulties, hyperesthesia at the surgical site, scar tenderness, and stiffness may contribute to the suboptimal results. Regardless of the underlying cause, it can be concluded that implementing an additional physiotherapy program has a positive effect on the intensity of CTS symptoms and pain in patients who were not fully satisfied with their treatment results during the first follow-up visit.

The percentage of patients experiencing persistent or recurrent CTS symptoms varies between 1% and 32%, according to various studies. In such cases, Gmainer et al. outline three potential treatment options: additional specialized physiotherapy, further diagnostic investigation, and reoperation. The rate of revision surgery ranges from 0.1% to 12%, with larger case series typically reporting revision rates around 5%. The choice of treatment depends on the nature of the symptoms. If recurrent symptoms arise, specialized physiotherapy or reoperation may be considered. Gmainer et al. state that in 94% of cases of recurrent CTS, the primary cause of revision surgery was the formation of hypertrophic, pathological scars, often linked to prolonged immobilization, poor hemostasis, hematoma formation, or improper hand therapy [8]. Similarly, Tung and Mackinnon report that recurrent symptoms are commonly due to pathological scar formation in the carpal tunnel area [9]. In cases of persistent CTS, further diagnostic evaluation and/or reoperation are considered, with the most frequent cause being incomplete division of the TCL or forearm fascia [8,9]. The emergence of new or different symptoms compared to those experienced prior to the initial CTR may suggest iatrogenic injury [9].

The use of specialized physical therapy is not always linked to persistent or recurrent symptoms. Dahlin et al. suggest that psychosocial factors may also play a role in the final treatment outcomes [10]. Our experience supports this, as some patients may seek to optimize their recovery during their period of work incapacity. Consequently, during follow-up visits, they often request a referral for additional physiotherapy treatments. Furthermore, some patients have unrealistic expectations, anticipating a complete resolution of all symptoms after CTR. However, this is not always achievable. This is corroborated by Louie et al., who reported that paresthesia may persist for up to nine months even after surgery [11].

Expanding on the topic of postoperative physical therapy, it is important to note that the range of physiotherapeutic methods following CTR is more limited compared to those utilized in preoperative conservative treatment. Multanen et al. reported that 67% of patients received some form of therapy within a year of CTR, although postoperative physical therapy was not universally implemented as part of the standard care protocol. A review of the literature reveals that the benefits of postoperative therapy remain a subject of debate. Despite high satisfaction with the surgical outcomes, a significant proportion of patients continue to seek conservative treatments even one year after the procedure [12]. Cantero-Téllez et al. observed that immediately post-surgery, the most commonly applied therapies include icing the operated area in the initial days (91%), neuromobilization techniques (81%), ultrasound therapy (66%), exercises as part of a home physiotherapy program (55%), finger and wrist flexor strengthening exercises after 15 days (41%), patient education (32%), sensory re-education (39%), electrotherapy (24%), and wrist immobilization (18%). Additionally, laser biostimulation is frequently used during the postoperative period following CTR [13].

The effectiveness of physiotherapy following CTR remains challenging to assess, as evidenced by the varying results in the existing literature. For instance, a study by Alves et al. demonstrated that patients who underwent low-energy laser biostimulation following median nerve decompression achieved better functional outcomes compared to a control group [14]. However, contrasting findings were reported by Sawan et al., who observed no statistically significant differences in pain intensity among patients who received laser biostimulation [15]. Electrotherapeutic treatments are relatively infrequently used after CTR. Nonetheless, studies by Gordon et al. suggest that such techniques can offer rehabilitation benefits, although these observations were based on electromyographic (EMG) results without evaluating key factors such as quality of life, grip strength, functional status, pain intensity, or muscle strength [16]. Additionally, Appelby et al. recommend the use of supplementary physiotherapeutic treatments with analgesic effects, such as ultrasound, massage, and gel compresses, particularly in cases of tender, painful postoperative scars [17]. Our observations support the idea that the selection of physiotherapy methods should be tailored to the specific symptoms or complications experienced by the patient, a strategy we have outlined in our research methodology.

During the course of our research, we were unable to incorporate some significant physiotherapeutic methods, such as extracorporeal shock wave therapy (ESWT) and high-energy laser therapy. Recent studies suggest that ESWT can be an effective treatment for pillar pain following CTR. For instance, Romeo et al. utilized ESWT in 40 patients experiencing pillar pain at least six months post-CTR. The treatment involved three sessions (2,800 pulses) performed weekly. The authors reported significant improvements in all patients four months after treatment [18]. Similar findings were reported by Turgut et al., who observed that ESWT significantly reduced pain intensity on the visual analog scale (VAS) in patients with pillar pain. In their study, the ESWT group underwent three sessions, one per week, each consisting of 2,000 pulses delivered at a pressure of 5 bar and a frequency of 5 Hz. The therapy was applied perpendicularly to the painful, tender, and swollen areas, targeting deep scar tissue. Initial sessions used a low energy flow density (0.03 mJ/mm²), which was gradually increased in subsequent sessions based on patient tolerance [19]. Additionally, in a randomized study, Haghighat et al. evaluated the effect of ESWT in 40 patients with pillar pain, compared to a control group, assessing conditions before and three months after treatment. Although the ESWT group showed a statistically significant improvement in pain relief on the VAS scale, no significant difference was found in hand function between the two groups [20].

When evaluating the effectiveness of CTR, it is important to note that patients with CTS associated with diabetes or previous wrist fractures generally have a less favorable prognosis compared to those with idiopathic CTS. Additionally, patients with normal electrophysiological test results tend to experience less favorable surgical outcomes than those with abnormal test findings. These patients are also more likely to encounter complications. Furthermore, axonal damage detected in electrophysiological tests is considered a poor prognostic indicator for surgical success [4].

The first limitation of the study was the inability to divide patients whose surgery did not meet expectations into a group undergoing and not undergoing specialist therapy. In our study, all patients underwent additional therapy. At this point, the question arises whether the observed improvement in these patients results from the effectiveness of physiotherapy treatments or is related, for example, to the passage of time. Certainly, the lack of a control group among patients receiving additional physiotherapy treatments significantly reduces the value of this study. The second limitation of our study was the lack of possibility to use specialist procedures that are currently very commonly used in postoperative therapy, i.e., ESWT and high-energy laser therapy. The third limitation that should be mentioned is that, as with any surgical intervention, there is a small subset of patients for whom the treatment outcomes are unsatisfactory, typically due to factors such as incorrect diagnosis or improper patient selection for the procedure. Another limitation is the fact that it is a single-center and non-randomized study.

## Conclusions

The implementation of additional specialized physiotherapy enhances the clinical outcomes of patients whose carpal tunnel surgical results were suboptimal or who encountered postoperative complications. The choice of physiotherapeutic techniques should be tailored to the specific symptoms or complications experienced by the patient.

## Appendices

**Table 5:** Levine Questionnaire – Symptom Severity Scale To calculate score, add together the scores for all 11 questions in part 1 and divide by 11

Part 1 of 2: Symptom Severity Scale (11 items)	1	2	3	4	5
1. How severe is the hand or wrist pain that you have at night?	Normal	Slight	Medium	Severe	Very serious
2. How often did hand or wrist pain wake you up during a typical night in the past two weeks?	Normal	Once	2 to 3 times	4 to 5 times	More than 5 times
3. Do you typically have pain in your hand or wrist during the daytime?	No pain	Slight	Medium	Severe	Very serious
4. How often do you have hand or wrist pain during daytime?	Normal	1-2 times/day	3-5 times/day	More than 5 times/day	Continuous
5. How long on average does an episode of pain last during the daytime?	Normal	<10 minutes	10 – 60 min continued	>60 minutes	Continuous
6. Do you have numbness (loss of sensation) in your hand?	Normal	Slight	Medium	Severe	Very serious
7. Do you have weakness in your hand or wrist?	Normal	Slight	Medium	Severe	Very serious
8. Do you have tingling sensations in your hand?	Normal	Slight	Medium	Severe	Very serious
9. How severe is numbness (loss of sensation) or tingling at night?	Normal	Slight	Medium	Severe	Very serious
10. How often did hand numbness or tingling wake you up during a typical night during the past two weeks?	Normal	Once	2 to 3 times	To 5 times	More than five times
11. Do you have difficulty with the grasping and use of small objects such as keys or pens?	Without difficulty	Little difficulty	Moderate difficulty	Very difficult	Very difficult

**Table 6:** Levine Questionnaire – Functional Status Scale To calculate score, add together the scores for all 8 questions in part 2 and divide by 8

Part 2 of 2: Functional Status Scale (eight items)	1	2	3	4	5
1. Writing	1	2	3	4	5
2. Buttoning of clothes	1	2	3	4	5
3. Holding a book while reading	1	2	3	4	5
4. Gripping of a telephone handle	1	2	3	4	5
5. Opening of jars	1	2	3	4	5
6. Household chores	1	2	3	4	5
7. Carrying a grocery basket	1	2	3	4	5
8. Bathing and dressing	1	2	3	4	5
